# The Significance of Touch in Pediatric Physiotherapy

**DOI:** 10.3389/fresc.2022.893551

**Published:** 2022-05-31

**Authors:** Marit Sørvoll, Gunn Kristin Øberg, Gay L. Girolami

**Affiliations:** ^1^Section for Innovation in Education, Norwegian Directorate for Higher Education and Skills, Tromsø, Norway; ^2^Department of Health and Care Sciences, Faculty of Health Sciences, UiT the Arctic University of Norway, Tromsø, Norway; ^3^Department of Clinical Therapeutic Services, University Hospital North Norway, Tromsø, Norway; ^4^Department of Physical Therapy, College of Applied Health Sciences, University of Illinois at Chicago, Chicago, IL, United States

**Keywords:** pediatric physiotherapy, children, clinical practice, embodiment, interaction, touch, handling

## Abstract

Change in theoretical framework over the last decades and recent research in pediatric physiotherapy, has created a debate surrounding therapeutic touch. What is the role of or is there a need for handling and hands-on facilitated guidance (facilitation)? Does it limit and/or interfere with children's learning and development? It is frequently argued that therapeutic touch represents a passive and/or static approach that restricts disabled children's participation during interaction and activity in clinical encounters leading to decreased home, school and community participation. Touch may even appear as coercive and controlling. In this context, therapeutic touch is largely associated with physical hands-on activities. However, therapeutic touch can also be understood as an intersubjective phenomenon that arises from a deep connection between movement, perception, and action. We believe the significance of therapeutic touch and its impact on physiotherapy for children has not been considered from this broader, holistic perspective. In this theoretical paper, we will apply enactive concepts of embodiment, sensory-motor agency, coordination, and emergence to explore the concept and importance of touch in physiotherapists‘ clinical face-to face encounters with children. We will frame the discussion within the context of the typical sensorimotor development of children from the fetal stage to birth on and into adulthood. Moreover, we will rely on biological, physiological, and phenomenological insights to provide an extended understanding of the importance of touch and the significance of touch in clinical practice.

## Introduction

Touch in physiotherapy is commonly assumed to be a vital component of clinical practice and is a core competence identified as therapeutic hands-on techniques (1). These techniques include specific sensory stimulation, tissue mobilization and handling (2, 3) and can convey interest, care and attention (1, 4, 5). Despite this view on the significance of therapeutic touch as an important element of clinical practice, tension has arisen around the use of therapeutic touch in pediatric physiotherapy, particularly concerning children with cerebral palsy (6–9). A recent systematic review (9) reported levels of evidence for interventions into green light (do it), yellow light (moderate evidence, assess the results), and red light (lack of evidence, don‘t do it). The interventions categorized as green light included any child-initiated problem-solving approach without touch from a therapist, for example constrained induced movement therapy (CIMT) and task specific training. In contrast, the therapeutic interventions categorized as red-light involved modes of therapeutic touch, for example neurodevelopmental therapy and sensory integration, which may give the impression that clinicians should avoid touch as a therapeutic mode. Additionally, during the last decades the theoretical frameworks of Dynamic Systems-, Perception-Action- and Motor Learning theories have dominated and guided the development of therapeutic interventions (10). These theoretical frameworks have contributed important knowledge concerning how different systems, the environment and the task can be integrated to improve function and the child‘s active participation. However, the latter has been used as an argument for hands-off approaches to promote participation during physiotherapy, at home and in the community, providing children to learn in natural environments while engaged in meaningful activities (6). Thus, touch as a clinical component in the conceptualization of physiotherapy interventions for children has been devalued and dis-acknowledged through both research and theoretical frameworks for clinical practice. Further, research claim that touch as a physical therapy modality is a passive and/or static phenomenon making the child an inactive recipient of treatment and obstructing engagement and motor learning (11–13). If this is so, how can physiotherapists during clinical encounters engage and interact with children with motor impairments that hamper their abilities to develop their motor competency and explore their environment? Could it be that therapeutic touch enhances motor competency and learning during clinical encounters as well as having an impact on what the child can do in broader functional and participatory contexts? Furthermore, to define touch solely as a passive and/or static physical phenomenon negates the importance and complexity of therapeutic touch in clinical practice. This includes the communicative aspects of touch, the ongoing dynamic touch generated interactions, the sense-making processes, bodily utterances, and body movements during therapy sessions.

Based on theoretical framework that speaks to the need for handling/touch as a therapeutic tool, we challenge the assumptions of touch as a solely passive and/or static physical contact issue between physiotherapist and child; one in which the child is viewed as an inactive recipient of passive and/or static touch and thereby the passive receiver of outside influences (14). A number of advances, especially in the field of child psychology, have contributed to a shift in thinking about child behavior and development from one of the children as passive receivers to one of children as social actors in their own lives (14, 15). Children as active agents and co-creators can also be translated to clinical encounters in pediatric physiotherapy.

Our perspective is founded in our experiences as physiotherapists who have worked with infants/children/adolescents with atypical development and neurological conditions, as academics with experience teaching therapeutic touch to graduate and post graduate physiotherapy students, and as researchers drawing on pragmatism and enactive theory to address the complexity of clinical practice. This includes therapeutic skills, intersubjectivity, and interactions between physiotherapist, children, and their families. Holding various positions and perspectives from a Norwegian and an American physiotherapy and research context, provide conceptual richness and methodological diversity to our arguments or discourse, highlighting taken-for-granted knowledge that underpins contemporary physiotherapy.

We propose an embodied approach (Table 1 - provides operational definitions of terms and concepts related to the embodied-enactive framework) that incorporates touch as a relational phenomenon. From an enactive perspective of touch, we will propose that touch in pediatric physiotherapy involves distinct aspects of physical contact, and what emerges during the dynamical processes of interaction between the physiotherapist, the child, and the parents. The issue then, is not whether touch is a passive and/or static approach, but rather touch as a required approach to engage and facilitate the child‘s movements and participation during clinical encounters. This may also apply to other areas in physiotherapy (5, 16, 17). In summary, from the enactive perspective, it is possible to advance the comprehension of touch as an intersubjective phenomenon which arises because of a deep connection between movement, perception, and (inter)action, and in turn should therefore be considered as a typical feature of clinical practice in pediatric physiotherapy.

**Table 1:** Defintions.

Autonomy:	Refers to living organisms, individuals, and groups of individuals that adaptively develop their capacities for flexible self-generated actions in relevant ways to maintain their organization and identities under precarious conditions. Precarious means that the individual's identity is affected by several processes creating change in either a positive or negative way. The principle of autonomy has roots in biological theory of autopoiesis (i.e., self-production) and applies to basic life-maintaining functions (e.g., the metabolic or immune systems) as well as advanced human actions (e.g., interaction with others and one's environment).
Dynamic systems theory:	A theory or approach that view non-living and living structures (e.g., individuals and groups of individuals) as complex self-organizing systems that display non-linear behavioral changes over time. Behavioral changes can occur in movement patterns in humans, or in interaction patterns in a group of individuals, and is caused by multilevel interactions between the various elements constituting these systems, as viewed from a third-person point of view.
Dynamic touch	The physiotherapist adapts and adjusts her use of own hands on the child‘s body in terms of direction, speed, strength, duration, range of touch and whether the hands should be on, off, or on again.
Embodiment:	Cognition is embodied actions. The body comprises a precarious network of autonomous self-producing/sustaining processes (e.g., metabolic, organic, cognitive, social) that relates to what we–our bodies–do in the world. When we act and engage with the world, a wide range of bodily processes, including cognitive experiences and sensorimotor and affective processes, occur simultaneously. The close connection between cognition and the action-oriented, experiencing body constitutes the body, the mind, and the brain as a systemic whole, an embodied cognitive system.
Emergence:	Relates to autonomy and sense-making, i.e., how we generate our identity and how we understand ourselves, others and our environment that are evolving and progressing properties and capabilities shaped by multilevel interactions within the mind-brain-body-environment synthesis.
Enactive theory:	Brings together phenomenological and dynamical accounts of how cognition evolves by highlighting the active role of a situated body in meaning- and world-making processes; the dynamics of a mind-brain-body-world systemic whole. The enactive account is constituted of five core principles: autonomy, sense-making, emergence, experience, and embodiment.
Experience:	Experience is what molds us as individuals. How we understand ourselves and others emerges from our embodied engagement with the environment. This embodied engagement consists of bodily movements and ongoing feedforward-feedback cycles (i.e., trials and errors) that give rise to experiences, particularly motor experiences. Experiences represent the process of learning and refining skills and thus facilitates cognition throughout the life span.
Minimal self:	An immediate consciousness of oneself as the subject of experience based on sensory processes such as proprioception.
Participatory sense-making:	Relates to social cognition through social interaction. Social encounters between two or more individuals produce forms of shared meaning-making through concrete actions, bodily movements, utterances, gestures, and speeches that could not be produced by either individual alone.
Passive touch:	The physiotherapist touches, moves and/or pushes the child around without any awareness what the child needs and requires to be an active participant in the situation.
Phenomenology:	The study of consciousness as experienced from the first-person point of view.
Sense-making:	Constitutes cognition, i.e., the generation of meaning through embodied interactions with other people and the environment. Based on our needs, desires, goals, and previous experiences we bring certain perspectives, concerns, and expectations to our encounters with others that shape how we perceive and understand ourselves and others and make sense of the world. Cognition thus includes processes that occur in-between an individual's mind-body and her environment.
Social cognition:	Following embodied approaches, social cognition involves the know-how that allows us to sustain interactions, form relations, understand each other, and act together.
Social touch:	An aspect of social cognition related to what happens in-between individuals as a basis of sense-making.
Static touch:	The physiotherapist places the child in a certain position and holds the child in that position

We will apply enactive concepts of embodiment, sensory-motor agency, coordination, and emergence to explore the concept and importance of touch in physiotherapists‘ clinical face-to face encounters with children. We will frame the discussion within the framework of typical sensorimotor development of children from the fetal stage to birth on and into adulthood; specifically, how typical children gain knowledge about themselves and how they interface with people and their environment via touch and interaction. The premise for communication, understanding and entering relationships with others as developing individuals into and throughout adulthood, is laid during early infancy (18), and for the purposes of this paper we will draw on examples from early infancy. Further we discuss how children developing atypically or those with neurological conditions have less ability to gain such knowledge and how this can guide the delivery of physiotherapy to this population. The latter includes important aspects of using touch to impart bodily knowledge and enhance adaptability and variability with these children. Moreover, we will rely on biological, physiological, and phenomenological insights to argue an extended understanding of the significance of touch in clinical practice. We begin with a brief introduction of the enactive concept of embodiment related to touch. Our purpose is not to elaborate on this concept in its full detail–this has been done in the work we refer to.

## Touch From an Embodied Enactive Perspective

Enactive theory is a synthesis of insights drawn from different fields of science including cognitive science, biology, dynamic systems theory, and the philosophy of phenomenology (19–21). One core idea is that the mind-brain is not the main driver for how we move and understand our body and surroundings, but the mind-brain is rather enacted and brought forward by the structure and organization of the body and its interaction with individuals and the environment. The mind-brain-body, and the environment thus connect and merge into a mutual dynamic relationship. Figuratively speaking, wherever and whenever connections arise, touch is involved in one way or another.

**Figure 1:**
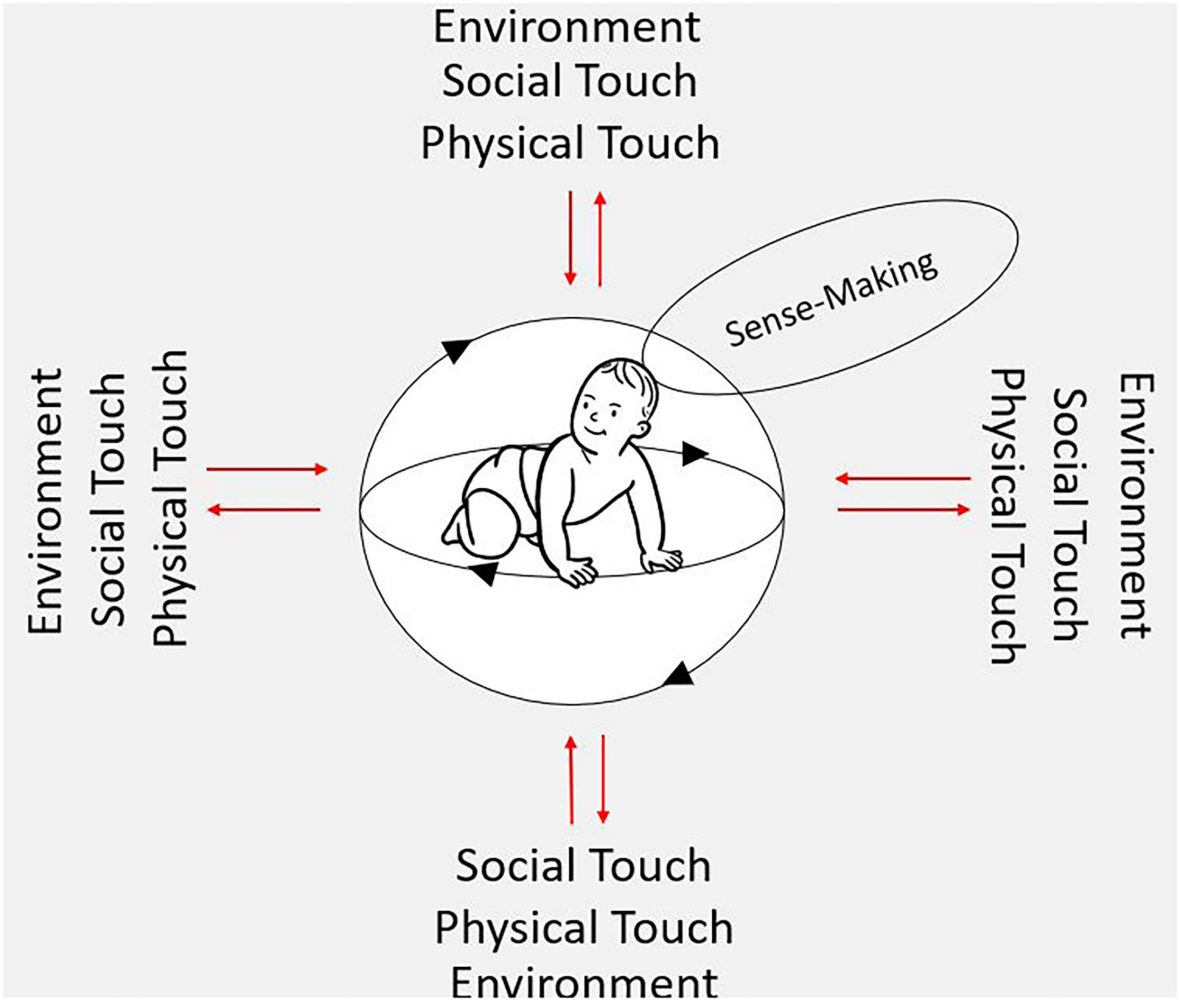
This figure is inspired by the work of of Di Paolor et al. (21). The two circles surrounding the infant illustrate an embodied, autonomous, self-generating individual (e.g., child) engaging meaningfully with her environment. The child always relates to her environment in terms of what it entails, where sense-making is the ongoing activity of creating value and significance. However, sense-making is not activity that “adds meaning” to the child's coupling to the environment but involves child in context where the sense-making co-emerges with the engagement and activities of that child. The eight red arrows represent the two-ways coupling between child and environment, in which social and physical touch are also part of the interaction.

Therefore, from the enactive perspective, the infant's body is not understood as an isolated and purely physical phenomenon but a living, sensemaking structure that is constantly in mutual interactions with its surroundings. As sensory inputs translate into motor actions, metabolic and homeostatic processes take place simultaneously. This is viewed as an ongoing mutual cycle between internal body processes, external environmental factors, and the effect of touch (see Figure 1).

**Figure 2:**
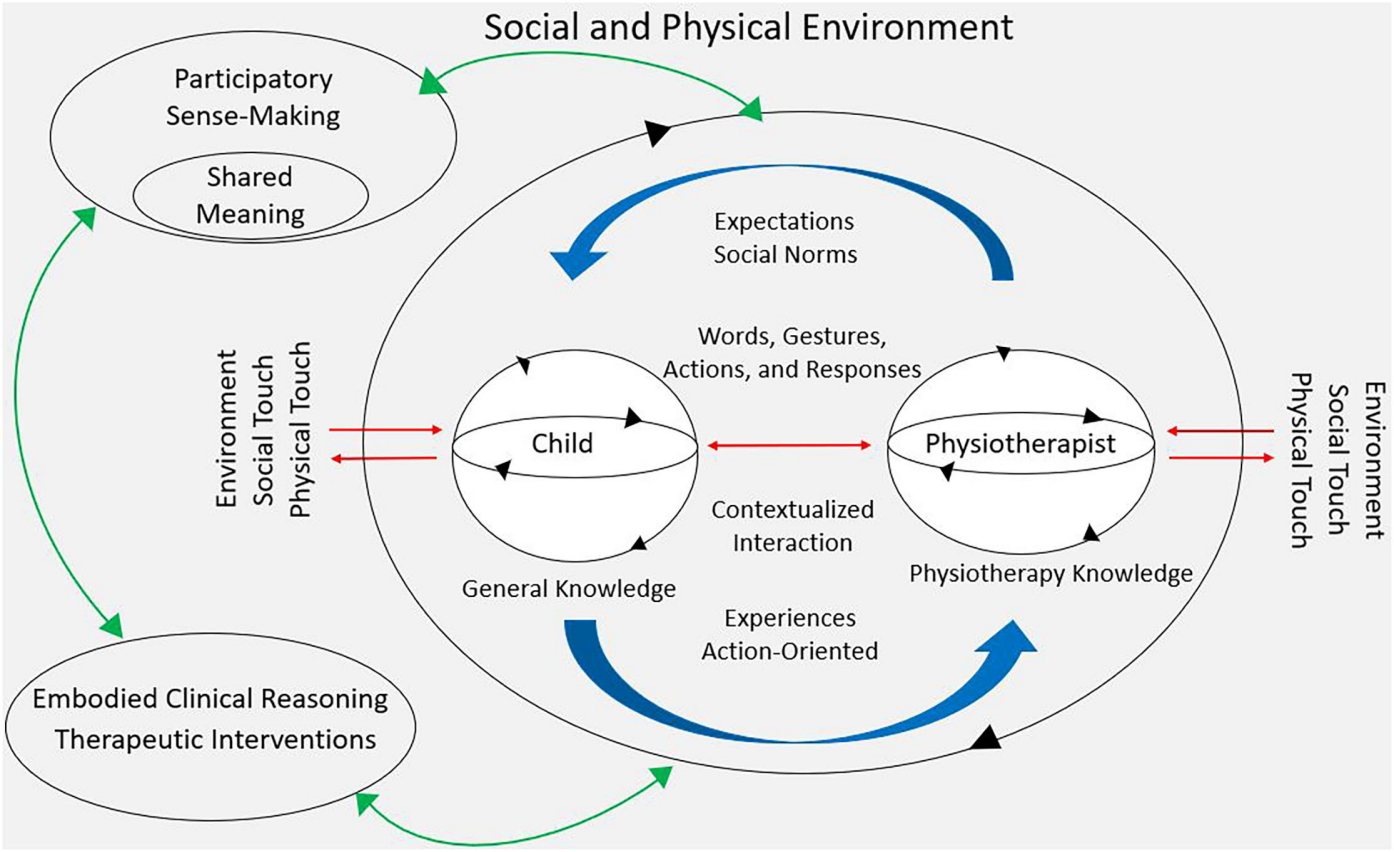
This figure expands on Figure 1 and is also inspired by the work of Di Paolo et al. (21) and the work of Øberg et al. (30), and illustrates the circular feedback between social environment, embodiment, coordination, and sense-making, i.e., the all-at-once ongoing relationships of Embodied Individuals (Child/Physiotherapist), Touch, Physical and Social Environment, Participatory Sense-Making, Embodied Clinical reasoning, and Therapeutic Interventions in a clinical setting. Environment is represented as a gray background intertwining all domains and comprises the affordances, opportunities and challenges the child and physiotherapist perceive during therapy. The two small circles illustrate child and physiotherapist; two autonomous self-generating individuals engaging meaningfully with each other and environment. The five straight horizontal red arrows represent couplings between child-and-environment, between physiotherapist-and-environment, and between physiotherapist-and-child, while the two thick blue curved arrows indicate the co-regulated coupling between the child and physiotherapist; the regulation each of them makes on their own couplings and on the other's. Hence, complexity increases when more people (e.g., parents, assistant, etc.) are added because each person is interdependently engaging meaningfully with the environment and with the other individuals during the therapy session. The big circle, which includes the child, the physiotherapist, and the intersubjective space between them, represents the interaction process and how it becomes autonomous, self-generates, and propels forwards as meanings and intentions are shaped and adjusted by the child and the physiotherapist. The three green curved arrows signify the emergence and co-creation of the whole, i.e., the mutually reinforcing relationship between all aspects.

Thus, the body is not limited to a sensorimotor system where sensory inputs go straight to the brain for processing. Rather, sensory inputs generate responses in the whole organism (body). For example, research shows that physical touch (i.e., baby massage) for 15-min daily sessions improves preterm weight gain and accelerates neurobehavioral development (22–24). Additionally, touch through visual stimulation (e.g., gaze and eye contact) initiates muscular and hormonal adjustments throughout the body, which create interoceptive sensations related to past experiences and guide the ongoing response to action (25, 26). This means the infant‘s various biological systems, such as the visceral systems, the musculoskeletal system, the neuromuscular system, the immune system, the circulatory system, and the endocrine system are influenced by each other (27). Furthermore, from an embodied enactive perspective, these systems are dynamic, autonomous, and self-organizing, which means they can grow, develop, and change their structure and functions based on exposure to and responses to internal or external circumstances (19, 27). For example, the musculoskeletal system in infants with moderate or severe cerebral palsy (CP) is often affected by delayed skeletal maturation and low bone density due to reduced weight bearing against gravity as well as decreased muscle length and imbalanced muscle activity (28). Hence, all dimensions of the child‘s living body, including its interaction with social and physical environments, contribute to how the mind-brain-body entity progresses and develops during early childhood and throughout life. In this context, the mind-brain-body entity goes beyond the outer surface of the body and transcends itself and partially incorporates with the environment. This incorporation arises from a high level of sensitivity, the ability to perceive and adapt, which extends the scope of pure physical touch alone (20). When two or more people are present, as in clinical encounters between physiotherapist, child and parents, their lived bodies are mutually coupled, facilitated through eye contact, gaze, facial expressions, voice, gestures, positioning, movements, and intentional actions. They enter a bodily state that incorporates the perceived bodies of others, and they learn about themselves and their partner from these experiences (20). According to De Jaegher and Di Paolo (29), this capacity to connect is essential for social understanding and shared “sense-making” processes, referred to as participatory sense-making and mutual incorporation (20). Pediatric physiotherapy then, can be seen as a unique form of participatory sense-making where sensitivity and different modalities of touch form an important basis to enhance child development (see Figure 2).

## The Significance of Touch for Child Development

Having presented the enactive concept of embodiment in the previous section, in this section we first show how embodiment connect to touch in early development i.e., how particularities of sensorimotor development, movements and perceptions are of importance for how the fetus and infant relate to their surroundings. Second, we show how touch is constituted through affordances of the environment and lastly, we link touch to social interaction.

### Emergence of Touch, Agency, and Embodied Self in Early Development

Touch is the very first sense to develop and evolve simultaneously, *in utero*, with the development of the nervous system. The human embryo (later fetus) receives continual multisensory touch inputs. Before the eyes and ears have developed, the human embryo responds to stimulation of the skin through touch and pressure from the amniotic fluid as well as the uterine wall and self-touch (31). It has been suggested that stimulation of the maturing sensory systems contributes to the structural and functional development of the nervous system (32, 33). As the embryo develops into a fetus, the capacity for regulating its interactions with the uterine environment begins to emerge. This regulation is an incipient emerging form of agency on a biological level and is coordinated by evolving sensory and motor capacities, which enable the fetus to discriminate, adapt, and recognize the boundaries of its own viability (34).

Therefore, fetal movements are an important underlying mechanism for co-dependency between fetal development and what the uterine environment affords. The earliest movements, beginning around 8 weeks, start in the fetal body, inducing shifting positions of the head and limbs floating in the amniotic fluid. This creates a series of self-produced responses, such as limbs that begin to flex and extend, or limbs that touch different parts of the body. As the fetus develops, the uterine space decreases, and the sensorimotor movement patterns go from self-produced to adaptive and self-regulating patterns (35). The ability to adapt is fundamental for sensorimotor agency; the capacity to act independently (34). Fetal movements, breathing, swallowing, and suckling are examples of sensorimotor agency that emerge from the interplay between the self-organization of the fetal body systems and touch from the uterine environment (34). This means the fetus creates meaning and is affected by its interaction with the uterine environment and is thus starting to become embodied and situated within its surroundings (34). This reciprocal touch between the fetus and mother is considered the earliest form of communication between two sensorimotor agents (34). Allover touch and movements are important for the development of sensorimotor capacities and thus the early emergence of an embodied self; the earliest form of cognition also referred to as “primordial sensitivity” (34).

The development of “primordial sensitivity” continues after birth. Studies on newborn imitation show that newborn infants, ranging from 1 to 71 h, can move their bodies in appropriate ways in response to interpersonal stimuli, such as imitating tongue protrusion, mouth opening and lip protrusion (36, 37). Once more the infant displays the capability being a sensorimotor agent. Hence, infants develop a body schema and a body image prior to birth which they utilize in communications and social surroundings through various touch modalities, postures, movements, firsthand experiences, and affordances in their surroundings (18). The body schema is a non-conscious system of sensory and motor capacities that enable the newborn infant to perform movements (18), while the body image provides the newborn infant with a primitive (non-conscious) proprioceptive awareness of its own body, for example the face (18). Body schema and body image are closely related to the sense of ownership and the sense of agency (18). Sense of ownership indicates the state of the body (18), for example an infant experiencing that a part of her body is moving as the physiotherapist moves her legs as part of assessing muscular tone. Sense of agency involves intention and the execution of actions (18), as when the infant reaches for a toy. These related perceptual systems structure the infant‘s consciousness; the proprioceptive awareness of minimal self-consciousness, an “embodied self” that is operative immediately after birth and continues to develop and function throughout childhood and throughout life. Thus, the “embodied self” is connected to bodily experiences, and the developing child is able to “map” the perceived bodily gestures, gazes and motions onto her own kinesthetic sensations (18). However, newborn infants who show poor sucking, poor muscle tone, atypical posture, atypical spontaneous movements and marked asymmetries may show reduced innate sensorimotor capacity. These interfere with the infant‘s ability to develop the competency and capacity needed for interpersonal communication and to fully participate in activities of daily life (38).

To explore touch in greater detail; from a neurobiological perspective, touch differs from the other senses (vision, taste, audition, and smell); it is not limited to a specific area of the body but is spread out over the entire body surface. Tactile (touch) stimuli of the skin activate afferent mechanoreceptive, such as fast-conducting myelinated A-beta afferents or slow conducting unmyelinated C-tactile (CT) afferents (39). The A-beta afferents respond to various forms of touch stimuli, while the CT afferents respond optimally to gentle and slow-moving touch at temperatures consistent with the human skin (39). Importantly, activation of CT afferents is related to positive affect and psychological pleasantness, and has shown to give rise to autonomic, neurochemical and behavioral responses (40). This has been substantiated by a Cochrane review of infant massage interventions that revealed dynamic (e.g., light stroking touch delivered from the first thoracic to the last lumbar vertebrae and in a reverse direction continuously for 5 min) rather than passive and/or static touch (e.g., hand placed on the dorsum of the infant covering the area from the first thoracic to the last lumbar vertebrae for 5 min) had a positive impact on weight gain and a reduction in length of hospital stay for preterm infants (41). Other studies have shown that short periods of dynamic touch (rather than passive and/or static touch) with a medium pressure and velocity between fast and slow produced increased levels of oxygen saturation in preterm infants (42). Research also shows that dynamic touch reduced heart rate in preterm infants and in infants as old as 9-months of age (42, 43) as well as increased attentional engagement, such as gaze shifts, and increased duration of visual attention (43). This suggest that dynamic touch help the infant exercise agency and communicate preferences through embodiment. Thus, speed, duration, excursion and rhythm of touch seem important and should be taken into consideration when doing therapy with infants developing atypically, or those struggling with attention and/or engagement during social interplay.

As previously mentioned, CT afferents are important for positive affect and psychological pleasantness and are described as the peripheral afferent path for awareness of social touch (44). In this context, the CT afferent route to the brain goes through the spinothalamic tract to a network of cerebral regions, for example the posterior insula, temporal cortex, and medial prefrontal cortex, which are important for the development and support of social cognition (39, 45). To illustrate, social touch, an aspect of social cognition, also involves interactions between individuals intertwined with the context; practices that happen in-between, such as the co-regulated embodied relationship between an infant, a caregiver (18) and/or a physiotherapist. Social touch can additionally be viewed from influences gained from the impact of the environment; social relationships, culture, contextual factors, and settings (18, 44). However, research suggests that there is a difference between typically developing children and children with neurological conditions when it comes to the ability to respond to social demands and constraints of interpersonal coordination (46). It is shown that typical children can coordinate their own body movements and rhythm to a present person during paired walking, while children with CP are not able to utilize such social facilitation to the same extent. They move in the same pattern without changes and variation. Their ability to perceive is challenged, and therefore, the physical presence of another individual alone is not sufficient for children with CP to adjust and coordinate their movements to the other. In contrast when a light physical touch is applied to their neck or head, they manage to change their movements with adjustments on a postural level and achieve more optimal walking pattern (46). This indicates the importance of addressing the CT afferents *via* touch in children with CP during physiotherapy to facilitate an awareness of their bodies with the intention to promote appropriate movements and the ability to utilize social facilitation.

### Accounts of Touch, Materiality, and Space in Infancy

During post-term sensorimotor development, the infant goes through different phases of tactile enquiry of her own body (touching herself, such as hand-to-mouth, hand-to-hand, hands-to-knees, and hands-to-feet movements), of materials (exploring toys and other objects that hold multiple tactile cues and textures: round, square, triangular, soft, hard, rough, etc.) and of spaces (moving from prone to supine position, from lying to sitting, moving along furniture, crawling up the stairs, etc.). One could say that the infant‘s body and the environment (materials and spaces) provide affordances for touch and movements. Hence, the sensorimotor and moving body allow the infant to explore, experience and penetrate deeper into the materiality of toys and other objects simultaneously getting to know her own body. The materiality of the physical world is not predetermined but emerges during a process of interaction and sense-making. Let us use the floor as an example of a tactile relationship the infant experiences. When the infant pushes her arms toward the floor to raise her head against gravity the floor affords a surface for pushing against, and due to its hardness, the infant simultaneously senses the resistance of the floor to her weight and force. In this regard, the floor provides a tactile passage for the infant's embodied self to move through. Moreover, in this position the infant body is touched in several ways, not only from the floor's hardness, but also by conditions such as the temperature as well as the light and smell in the room. Even a potential draft along the floor provides a condition that sparks awareness. As the infant moves further in a particular direction, for example by rolling, different parts of her body touch the floor in new ways. The introduction of new movements causes great shifts in how the infant relates to the floor and her own body, and sense-making arises in the interface between the infant body and the surface of the floor. However, infants with atypical or neurological conditions are often challenged to generate these types of experiences on their own. They lack the variability of movements, and their movements reinforce stereotypical and non-optimal movement patterns. To promote various motor experiences and self-movements in children with functional activity limitations, it seems necessary to consider what therapeutic approaches are the most effective ones to facilitate movements through the desired task. Promoting self-movements and positive motor experiences to enhance motor learning in these children, it seems important to grade and direct the therapeutic strategies to provide proprioceptive, tactile, and kinesthetic information relevant for the task.

Kinesthetic sensations are a non-conscious system and inform us about the position and movement of our own body, in particular the position and movement of the limbs (47). Sheets-Johnstone (47) highlights the importance for the child to experience various tactile-kinesthetic feelings during movements, for example in relation to play, which in turn implies the ever-present modality of touch. When, for example, the child reaches for a toy or brings the spoon to the mouth, the kinesthetic sense provides her with an internal knowing about the arm's position in relation to the trunk based on proprioceptive inputs and feedback from receptors located in the muscles, joints, ligaments, and tendons (18). Touch, movements, and kinesthetic feelings provide the child with a dimension of corporeal powers, also referred to as the “I-can” and “I-cannot” (47). This means the child, through touch and movement explorations, generates (and regenerates) meaning by discovering tactile-kinesthetic and spatiotemporal possibilities of own body. Research indicates when a typically developing infant begins reaching movements, a clear direction of arm movements is lacking, and the infant produces nonproductive actions throughout the body (48). Gradually, as the infant accidentally swipes, bangs into or brushes against a toy, the infant starts to create head and arm movements that become more task-related, efficient and effective over time (48). However, this may be difficult to achieve for children with moderate to severe spastic CP as they often lack the ability to create reaching movements due to increased muscle tone in the arm flexors and supinator.

Further, the typically developing infant discovers her own arm-hand possibilities through spontaneous non-task related arm actions, but at the very moment the hand touches the toy, the infant starts to coordinate own arm actions according to the toy's characteristics. For example, a toy attached to a string and moves when touched, becomes an important sense-making process for the infant as the arm “coordinates-to” the toy's movements in a synchronized-desynchronized flow, also referred to as unidirectional incorporation (20). “Coordination-to” seems to drive developmental changes as the infant produces more task-related arm actions (48). Further, as the infant goes from touching to grabbing the toy and then uses it for new purposes (banging the toy against the floor or other toys), you may say that the toy becomes increasingly integrated into the infant's developing motor schema and the infant's arm-hand and toy co-constitute each other. What the infant senses by her touching hand guides what she feels by her movements simultaneously as the shape and movements of the toy guides the hand's touching. All these processes, initiated by various modalities of touch, movements, and tactile-kinesthetic feelings supported by vision, are profoundly important for sensorimotor development in children and human potential in general. More importantly, it implies the significant role physiotherapists have treating children with atypical motor development to emphasize facilitation of the child‘s own movements in accordance with the child‘s self- induced motor initiative and potential.

### Social Interaction: Incorporation, Coordination, and Emergence During Childhood

Touch between infant and caregiver is considered important for sensorimotor, emotional, and cognitive development in early childhood (49), and becomes a channel for communication, representing a source of sensory feedback to the infant. This communication differs from the previously mentioned unidirectional reactive handling of objects. As opposed to the child's attempt to “coordinate to” an object, an interaction between a child and her caregiver is a social encounter, in which they reach out to each other through their lived bodies as they perceive the other simultaneously and being perceived by the other. During this reciprocal process, in which intentions are generated, transformed, and expressed in the very process of interaction, the child and her caregiver may “coordinate with” each other (20). This also applies for clinical encounters between a physiotherapist and a child. The coordination and synchronization will emerge in various degrees depending on the kind of interaction the physiotherapist and child have. However, as social interactions described in general terms by Fuchs and De Jaegher (20), therapeutic interactions always have a bidirectional and interactive character, affecting who the participants in the clinical encounter are allowed to be and become for each other in the very situation. The child as well as the physiotherapist are co-determined with the other through touch, gaze, attitude, posture, and movements where behaviors contribute to regulate each other and the co-constitution of the development of the situation. Engagement, force, and velocity in the interaction process may override their individual intentions and the therapeutic interaction develops an autonomy of its own (20, 29). What happens “in-between” becomes the origin of the operative intentionality of the child and the physiotherapist and a basis for development of a common sense-making (20). This implies that neither the child nor the physiotherapist has complete control over themselves, the other, or the situation while common sense-making emerges (20). In other words, in clinical encounters the interpersonal dynamic bodily dialogue between the child and her physiotherapist and the process of interacting itself becomes relevant for what is allowed to emerge in each situation. Hence, different modalities of touch are interactionally organized and embedded in the clinical context and the social interplay between the physiotherapist and the child.

Therefore, even though newborn infants have innate sensory and motor capacities enabling them to connect and communicate with others, the infants are simultaneously dependent on the impact of others to touch, perceive and understand the infants' bodily cues and expressions throughout childhood. It has been shown that children in institutional care, who often receive very little touch, show cognitive impairment (50, 51), and that children of depressed mothers compensate for the lack of their mothers' touch with increased self-touch behavior (52). Children are vulnerable individuals which means that special consideration must be given to safeguard their sense of agency in the process of interacting. This gives rise to a tension, on the one hand balancing the child‘s vulnerability and on the other hand enhancing the child as an active participant/agent.

In a dynamical process of interaction and coordination a co-continuity of negotiations will take place. Rhythm, synchronization and co-variation of bodily expressions and movements may occasionally attune, meaning that social interplay consists of both matches and mismatches (20). Research on maternal-infant interactions reveals that up to 30% of interaction time is spent misperceiving each other's expressions (53). However, when mismatches occur, subsequent successful repairs, where synchrony between the child and mother is regained, are shown to be important to provide the trust necessary for the dyadic interplay and to enhance development and sense of agency in children (20). This obligates the caregivers to be responsible for successful interplay in encounters with children and reinforces the importance of physiotherapist's responsibilities in clinical encounters.

To provide an extended understanding of touch we have, in this and previous sections, discussed enactive concepts of embodiment, sensory-motor agency, coordination and intersubjectivity within the context of the typical sensorimotor development of children. In the following we will apply these insights to physiotherapy.

## Touch and Practice in Pediatric Physiotherapy

Pediatric physiotherapists are autonomous practitioners who possess the necessary competence and clinical skills to provide assessment and treatment of infants, children, and adolescents with a variety of conditions that impact development. Their recognition of the enactive framework, contributing to the conceptualization of touch as an intersubjective phenomenon, is necessary to understand the importance of embodiment for the emergence of actions, interactions, and sense-making processes in clinical encounters. From this perspective, the embodied knowledge of the physiotherapist becomes central not just for the process of interacting, but also for the development of clinical skills, how the therapist perceives herself, and her contributions to co-create optimal changes in the child's function, movements, and participation. The physiotherapist‘s embodied sensitivity becomes a source of impact on her own body as well as on the child‘s body. This implies that the physiotherapist‘s embodied sensitivity includes different modalities of touch comprising aspects of both physical and social touch. Consequently, from this perspective, movement analysis and clinical reasoning, which are considered core elements of clinical competence, goes beyond a purely cognitive activity, and includes intersubjective communicative practices through incorporeity (30).

To be clear then, embodied incorporeity is not just a matter of knowing and performing methods and techniques but rests on how the physiotherapist perceives the intended meaning of the child‘s gestures, postures, movements, expressions, and the timing and the emotional attunement during reciprocal embodied communication with the child. The physiotherapist‘s movement analysis and clinical reasoning emerges during the sensorimotor processes of “coordination-to-and-with” the child. There are various choices to be made both by the physiotherapist and by the child. However, as previously mentioned, the physiotherapist has the primary responsibility for the interaction process, which implies the physiotherapist should be sensitive and aware of the child‘s vulnerability in the situation (14). The child is not a predetermined structure, but an emergent, experiencing, and sense-making individual that shapes and is being shaped by interactions with the physiotherapist. As such, the autonomy and the vulnerability of the child is continuously at stake during therapy. This underscores the importance of the physiotherapist's ability to adapt the therapeutic process to a complex dynamic, i.e., the material selection and positioning of equipment (toys, mats, furniture, etc.), the tasks, and the coordination of the physiotherapist's body, movements, and sense-making in accordance with the child's movements, bodily expressions, and sense-making. Hence, touch is more than hands-on intervention (4, 30, 54). In a broad sense touch is a path for social embodied communication (1, 55), which strongly facilitates the therapist's adaptations and adjustments according to the infant's expressed needs during therapy (4, 56). For example, if the physiotherapist perceives the child‘s gaze turns toward a toy in the distance, ideally, she would understand and act on the child‘s cue by bringing the toy into the therapy session. In another scenario, the infant may yawn, show skin color changes, or the quality of movement, muscle tone and/or activity level may change during the therapists handling of the infant indicating an embodied expressed need for a change or a break in the session. The fully attentive and sensitive therapist will respond and provide the needed adjustments or break.

Various therapeutic approaches constitute an integrated powerful repertoire enabling the physiotherapist to act in response to the child's choices, sensorimotor challenges, and needs. It is not a matter of whether the therapist should be hands-off or hands-on, but how the therapist blends and integrates aspects from different approaches into the physiotherapist's clinical performance to promote the child‘s potential. However, each approach (hands-on and hands-off) may separately represent a reductionistic approach – a “coordination-to” the child as an object – if they are only performed as pure procedure or routine without variations and adaptations to the context, situation, and the child. In that case, the child will be placed on the periphery of the sense-making processes during clinical encounters. Therefore, when the physiotherapist aims to promote movement skills in children the therapist must possess an embodied awareness that enables the integration of movement analysis, clinical reasoning, and treatment measures to promote the child's engagement and potential.

Specific structural changes take place in the central nervous system neuronal networks in response to activity and functional demands (57). As previously described, typically developing children display a variability and adaptability in their movements, while children with neurological conditions, for example children with CP, are disadvantaged in their ability to achieve adequate levels of physical functioning because their sensorimotor, musculoskeletal, neuromotor and the cardiorespiratory systems were not fully developed before the brain injury occurred. Atypical development is manifest as various compensations, clinically observed as atypical muscle tone, deformities, asymmetries, and immature postural control (10). This implies that these children often require relevant positioning and a hands-on approach that facilitates movements and leads to co-creating the child's sense of agency and ability to move herself in variable and adaptive ways. Taking the enactive perspective, communicative practices involving positioning, touch and facilitated guidance through hands-on approaches should be done in a way that invites and enables the child to actively participate in controlling the movements to achieve shared agency (joint action) and participation in the situation. Therefore, it is not solely about the physical touch itself, but *how* and *where* we as physiotherapists touch the child during the interplay for co-creation of meaning. Touch in this matter becomes a way of mutual communication where the pressure, velocity and direction of physical touch is adapted to the child‘s initiatives, bodily expressions, and responses. Therefore, the therapist's hands-on facilitation (touch) is not merely about a passive and/or static physical touch or stimulating the child's body and movements, but a means of creating an interplay between the body, mind and brain to support the child's embodied self (4, 18). Simultaneously as the infant responds to the physiotherapist's hands-on movements, the physiotherapist may co-respond to the infant's bodily answers. Being hands-on also implies knowing when to be hands-off to foster the child's ability to take over and perform the movement by herself. During this participatory sense-making process, the physiotherapist and the infant create we-space, a mutual incorporation, in which the mutuality of the sense-making is strongly co-coordinated, and the bodily actions and interactions are strongly co-synchronized. As such, one could argue that the therapist's successful facilitated guidance goes beyond targeting the infant's motor skills and touches the very core of the infant's embodied self. That is, how the infant perceives and represents herself at the most minimal, pre-reflective level. In fact, supporting the infant's identity, who she is and is allowed to be at this very moment during this therapy session.

Let us use the example of a 4-month-old infant having difficulties with head-lifting while prone on elbows. Head control is one of the earliest motor developmental skills and considered to be an important milestone and the foundation for more advanced motor development (58). When the physiotherapist guides the infant's movements, for example, by placing hands on the infant's gluteal muscles with a gentle pressure in frontal and caudal direction, the infant may respond by taking more weight on the forearms followed by a head lift. Awareness of the importance of touch implies the therapist knows a change in the hand position, the amount of pressure and/or introducing a toy to engage the infant will help her sustain the new and more upright head and trunk position. The physiotherapist may also choose to let go of the grip when she experiences and perceives that the child is able to maintain the position of the head and body without the use of facilitated touch. The infant‘s spontaneous exploration of movement possibilities is enabled by the physiotherapist‘s sensitivity to the child‘s embodied manifestations and adjustments.

Touch is reciprocal; the physiotherapist and the child touch and are being touched by each other, alternating in acting and being acted upon. According to the enactive perspective, such sensorimotor processes through bodily interaction between the physiotherapist and child, will provide the therapist insights through somatosensory, proprioceptive, or kinesthetic feelings of movement. A mutual bodily resonance may gradually develop within the dyad; the therapist's body extends to include the body of the child, and the body schemas of each blend into one system, known as mutual incorporation (20). Additionally, interacting with numerous children in various contexts allows the therapist to gain multiple experiences in attuning to the bodies of diverse children while addressing their embodied selves, including their body schemas. Consequently, the physiotherapist's kinesthetic pattern recognition may be enhanced contributing to the development of advanced therapeutic skills which in turn helps them gain enhanced bodily knowledge. Through observation and handling of children in different contexts, the therapist develops an embodied therapeutic skill that enables her to intuitively anticipate the child's intention to move (59–61).

As described, skilled hands-on intervention requires the therapist to be in the moment [i.e., reflection in (inter)action], which heightens awareness and responsiveness and the ability to effect handling changes in anticipation of the child's bodily expressions and responses (60, 62). Hence, the handling/touch is not passive and/or static but active and dynamic; an interaction that unfolds in such a manner that the child and the physiotherapist can incorporate new bodily experiences contributing to the development of body schemas of the child as well as the physiotherapist. This has been substantiated by research concerning basic neurobiological consequences of the use of simple tools, e.g., holding a stick, (63–65), showing that dynamic use of the stick not only extended individuals reaching space, but also incorporated the stick and the reaching space into a plastic neural representation in their body schemas. In contrast, passive use of the stick, i.e., just holding it in their hands, did not have the same modulating effect on the body schemas. Therefore, an example of translating this research on the therapist's use of touch can be seen when the physiotherapist positions her body and adapts hands dynamically to the shape, size, and responses of the child's body during interaction. We propose that the therapist's body schemas adjust and attune to the circumstances of the child in context of the activity. Likewise, if we look at this from the perspective of the impact of the child, offering her a tool in a functional context would together with the therapist handling allow the child to develop a sense of agency and understanding of her “embodied self.”

In summary, touch is not only the use of hands but blends into the social aspect and is intertwined with environmental touch; physical contact with objects (toys, furniture, floor, etc.) or affordances provided by objects in the environment (the ball affords to be picked up, the stairs afford to be climbed, etc.). In every clinical encounter, the physiotherapist must consider treatment strategies and approaches in relation to what is evolving in the therapy session, blending various approaches to meet the child's needs and challenges. This implies the therapist must possess embodied skills that allow her to make changes and adaptations throughout each treatment session. Such embodied skills enable a process of interpersonal coordination of movements as part of the sense-making processes, mutual incorporation and what develops “in-between” the therapist and child in every therapy session.

## Final Remarks

We have elaborated on the concept of touch in pediatric physiotherapy within the framework of typical sensorimotor child development and the theoretical framework of enactive embodiment and intersubjectivity. We have highlighted the significance of touch in pediatric physiotherapy by drawing on examples from early infancy. However, the concepts presented in this manuscript emphasize how the many modalities of touch are significant for every clinical encounter whether it concerns infants, children, or adolescents. This constitute a basic understanding that also can be translated to clinical encounters with adults. We propose touch is both physical and social and blends into pediatric physiotherapy through co-regulative interaction processes. This necessitates the physiotherapist be aware of and sensitive to the child in context of the treatment, attuning and positioning her body to the child's initiative, body, and (inter)actions. What the child experiences is important for what the child learns. Adaptive learning environments encouraging the child to unrestricted self-exploration of own movement repertoires are of importance. However, children developing atypically often require individualized support, facilitation, and handling to help them engage, gather information from the environment and self-explore own movements. Positioning and coordination of bodies, movements, and touch between the child and physiotherapist are therefore critical aspects of actions and achievements in physiotherapy. Looking at therapeutic touch through the lens of the concepts of enactive embodiment, intersubjectivity and child development, we have illuminated the complexity of clinical practice. Through our elaborations we have shown that the complexity of touch is nothing to avoid but should be embraced as it appears basic to clinical practice in pediatric physiotherapy.

## Data Availability Statement

The original contributions presented in the study are included in the article, further inquiries can be directed to the corresponding author.

## Author Contributions

MS and GKØ were responsible for the conceptualization, preparation, and first draft of the manuscript. Additionally, MS drafted and prepared the figures. All authors contributed to the review and revision of the manuscript, approved the submission, and are accountable for all aspects of the work.

## Conflict of Interest

The authors declare that the research was conducted in the absence of any commercial or financial relationships that could be construed as a potential conflict of interest.

## Publisher's Note

All claims expressed in this article are solely those of the authors and do not necessarily represent those of their affiliated organizations, or those of the publisher, the editors and the reviewers. Any product that may be evaluated in this article, or claim that may be made by its manufacturer, is not guaranteed or endorsed by the publisher.
